# Reconstruction strategy in isolated complete Cryptophthalmos: a case series

**DOI:** 10.1186/s12886-019-1170-6

**Published:** 2019-07-31

**Authors:** Zhaochuan Liu, Binyu Xie, Yang Li, Jingwen Ding, Dongmei Li

**Affiliations:** Department of Ophthalmology, Beijing Tongren Hospital, Beijing Ophthalmology and Visual Science Key Lab, Capital Medical University, No.1 Dong Jiao Min Xiang Street, Dongcheng District, Beijing, 100730 China

**Keywords:** Complete cryptophthalmos, Reconstruction, Orbital development

## Abstract

**Background:**

The present study sought to introduce clinical characteristics and stepwise surgical strategies of isolated complete cryptophthalmos, a rare, congenital ocular anomaly.

**Case presentation:**

Retrospective, noncomparative, clinical study. Six patients with isolated complete cryptophthalmos were diagnosed at the Beijing Tongren Hospital between 2010 to 2018. The presentation age of patients ranged from 1 month to 68 years. This study includes two males and four females, and involvement was noted to be bilateral in two cases and unilateral in four cases. According to orbital CT scan and ocular CDI results, two patients were combined with ocular cyst. Reconstruction surgeries were performed in three patients, involving the eyeball enucleation, creation of fornix, eyelid reconstruction with skin flaps/amniotic membrane, and implantation of prosthesis. Besides, implantation of hydroxyapatite was performed in one pediatric patient to promote orbit development. Good outcomes in terms of cosmetic satisfaction were achieved in all patients during follow-up.

**Conclusions:**

Surgical intervention could only improve the cosmetic appearance in isolated complete cryptophthalmos. The surgical strategies may be planned to use three-stage approaches described in this study. Meanwhile, orbital development must be taken into consideration in pediatric cases.

## Background

Cryptophthalmos, first described by Zehender in 1872, is an extremely rare, autosomal recessive ocular disorder and may occur in isolation or as a part of Fraser syndrome. [1] Cryptophthalmic eyes, representing a fundamental failure in ocular development, have a very poor prognosis for visual function and have received special attention among oculists. [2] In 1969, Francoise classified the clinical findings of cryptophthalmos into three subtypes: complete, incomplete, and abortive form (see Table 1). [3] In complete cryptophthalmic patients, the upper and lower eyelids are replaced by a sheet of skin running from forehead to cheek. The eyebrows are absence or poorly developed, and eyelashes cannot be observed. [4] In addition, the ocular structures are grossly disorganized and may also present as a cyst . [4, 5]

**Table 1:** Classification and Ophthalmic Features of Cryptophthalmos^2,3^

Subtype	Characteristics
Complete	Failure of formation of the lid folds and globe results in skin extending from the brow to the cheek without identifiable adnexal structures; Ultrasound may identify a vestigial ocular structure or cyst within the socket
Incomplete	An ill-defined upper eyelid is completely fused, often over an abnormally developed globe and a keratinized cornea
Abortive	The upper lid is absent with a fold of skin extending from the forehead with variable degrees of adhesion/fusion to the underlying cornea; There is a normal lower lid (although often elongated) and relatively normal lower cornea. Variable degrees of microphthalmia may coexist

Rehabilitation of cryptophthalmos presents an extremely challenging task for ophthalmologists. [2] In most complete cryptophthalmic cases, there are no visual potential and the primary objective of surgical intervention is to improve cosmetic appearance. [2] In pediatric patients, the development of the orbit must be highly-considered. Therefore, stepwise methodical approaches are requested, which differs according to the severity of the abnormalities as well as the orbit development. Our previous study focused on the surgical management of cryptophthalmos and summarized our experience in managing abortive cryptophthalmos with a one-stage reconstructive method. [6] The aim of current study is to introduce our experience in the clinical observations and stepwise reconstructive strategies which achieved satisfactory cosmetic results.

## Case presentation

The medical records of patients with isolated complete cryptophthalmos who were referred to Beijing Tongren Hospital between 2010 and 2018 were reviewed. All patients were examined and managed by the same oculoplastic surgeon. Data were documented including the age at presentation, gender, consanguinity, family history, clinical presentations, surgical interventions, and outcomes. Patients’ images were taken for confirmation of the physical findings. In addition to ophthalmologic examination, patients received ocular CDI and orbital CT scan preoperatively. The study adheres to the tenets of the Declaration of Helsinki for research involving human subjects and was approved by the ethics committee of Beijing Tongren Hospital. Written informed consent from patients or their parents was obtained for publication of potentially identifying details as well as the images in this case series.

**Fig. 1:**
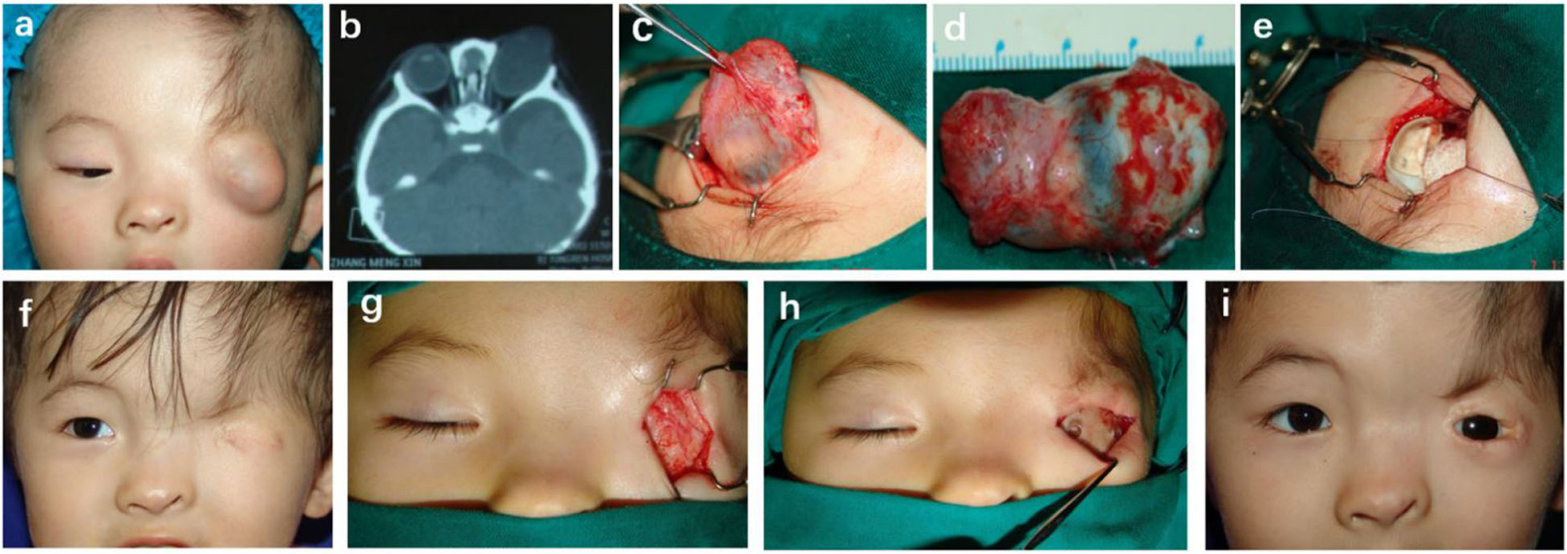
**a** Preoperative patient with complete cryptophthalmos and a cystic swelling arising from the left orbit. **b** CT scan shows an enlarged, bulging globe with increased anteroposterior dimensions in the right side. **c** Intraoperative photograph shows a large cystic globe. **d** Enucleation of enlarged cystic eyeball. **e** Implantation of 21.0 mm HA and banked sclera. **f** One year after the first stage surgery. **g** Construction of eyelids and socket using full-thickness skin graft. **h** A plastic eye shell was placed to maintain the conjunctival sac. **i** 1.5 years after the second stage surgery with prosthesis

**Fig. 2:**
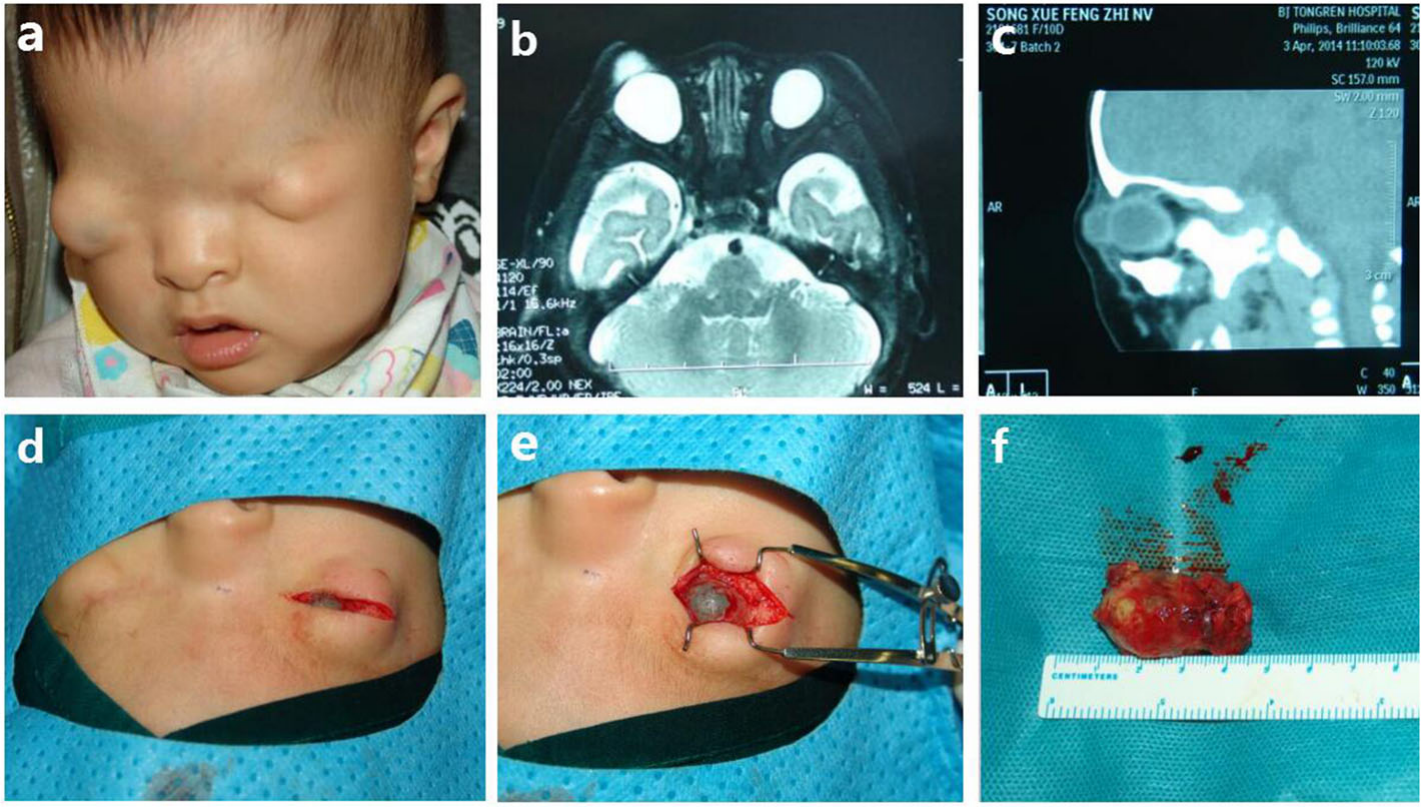
**a** An eleven-month-old girl with complete cryptophthalmos bilaterally and proptosis in the right side. **b** MRI shows an enlarged, bulging globe with anterior cyst in the right side. **c** CT scan displays right eye proptosis with anterior cyst. **d**-**e** Intraoperative photograph shows a large cystic globe. **f** Enucleation of enlarged cystic eyeball

**Fig. 3:**
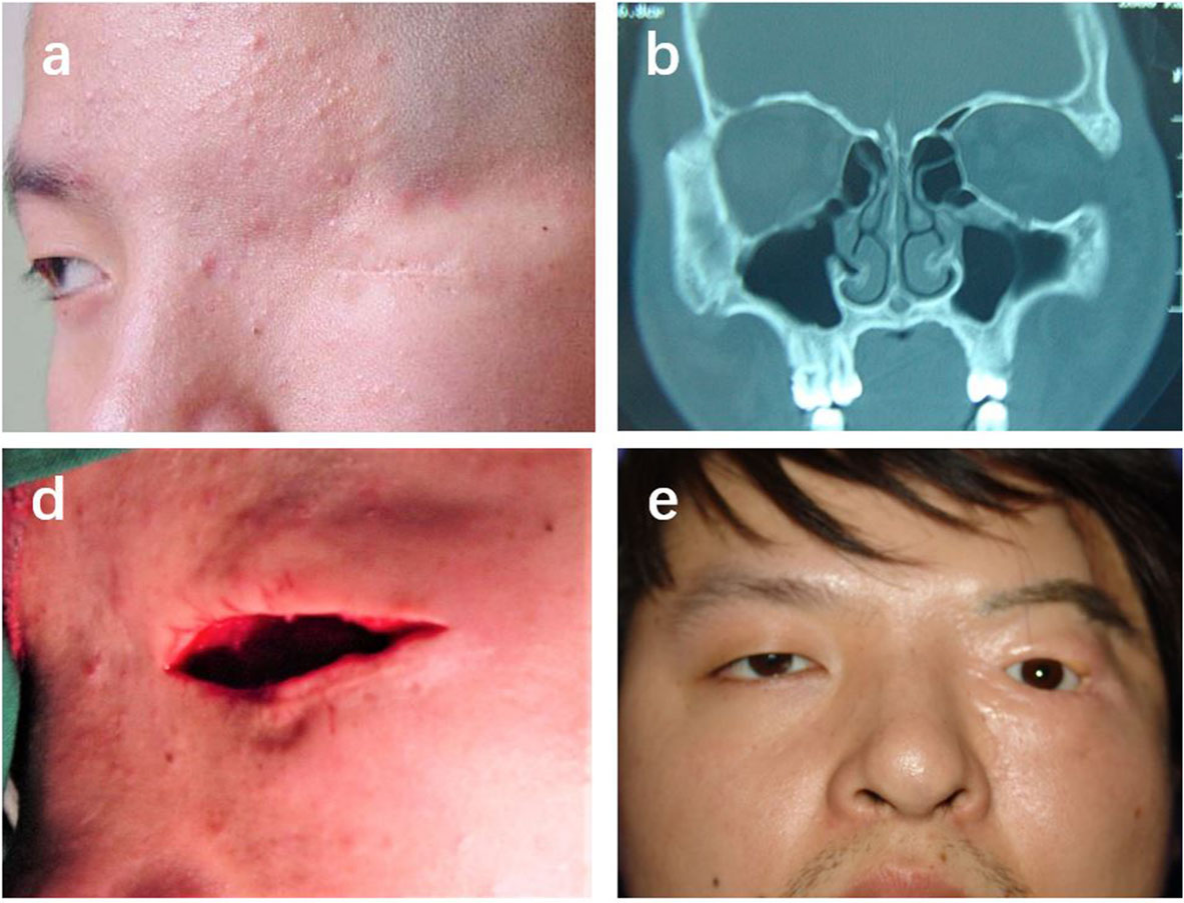
**a** Preoperative patient with left complete cryptophthalmos. **b** CT scan examination. **c** Reconstruction of the socket using full-thickness skin graft. **d** Two years postoperative view with prosthesis

**Fig. 4:**
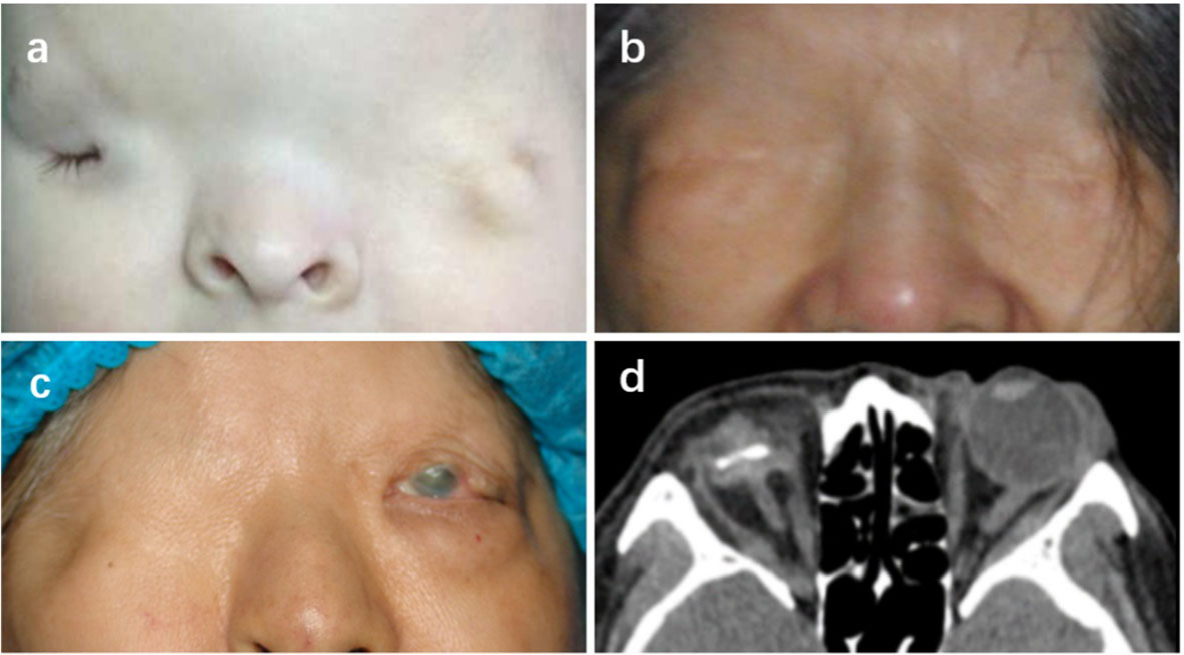
**a** Case 4, one-month old boy with complete cryptophthalmos OS and incomplete cryptophthalmos OD. **b** Case 5, the first sibling with bilateral complete cryptophthalmos. **c** Case 6, the second sibling with complete cryptophthalmos OD and abortive form OS. **d** CT scan of Case 6 displayed severe disorganized intraocular structure OD

As can be seen in Table 2, this series include eight eyes of six patients, and involvement was noted to be bilateral in two cases and unilateral in four cases. The presentation age of patients ranged from 1 month to 68 years. According to orbital CT and ocular CDI results, two patients were combined with ocular cyst, while others presented disorganized intraocular structure. Besides, siblings were affected in two cases. Surgical intervention was recommended only if the patient requested for cosmetic improvement. Reconstructive surgeries were performed in case 1–3 (see Figs. 1, 2 and 3 respectively), involving the eyeball enucleation, creation of fornix, eyelid reconstruction with local skin flaps/amniotic membrane and implantation of prosthesis. Unfortunately, patients in case 4–6 disagreed with surgical intervention and no surgery was performed. (see Fig. 4).

**Table 2:** Patients with Isolated Complete Cryptophthalmos in Beijing Tongren Hospital (2010–2018)

Pt.No.	Sex	Age at Presentation	Ethnic Origin	Parental Consanguinity	Associated deformities	Laterality	Ocular Cyst	Visual Potential	Surgeries
1	F	4 months	Asian	No	Bifid nose, aberrant hairline	OS	Yes	No	Yes
2	F	11 months	Asian	No	Absent eyebrow	OD	Yes	No	Yes
						OS	No	No	No
3	M	24 years	Asian	No	Aberrant hairline, absent eyebrow	OS	No	No	Yes
4	M	1 month	Asian	No	Incomplete form OD, aberrant hairline, absent eyebrow	OS	No	No	No
5	F	61 years	Asian	No	Aberrant hairline, absent eyebrow	OD	No	No	No
						OS	No	No	No
6	F	58 years	Asian	No	Abortive form OS, absent eyebrow	OD	No	No	No

## Case 1

A four-month-old girl born with absent eyelids, anterior hairline anomaly on the left side, and her eyebrow was replaced by a downward sweep of the frontal hairline and a mass which was gradually increasing in size from the left orbit. (Fig. 1a) The right eye, eyelids and systemic examination revealed no abnormality. During pregnancy, her mother had no history of illness, trauma or exposure to toxic agents or radiation. The child was diagnosed as complete cryptophthalmos OS. On examinations of CT scan (Fig. 1b), there was a cystic swelling arising from the left orbit and the eyeball cavity was communicating with the cyst.

The first stage of surgical interventions included enucleation of cystic eye and hydroxyapatite (HA) implantation were performed. (Fig. 1c-e) Following horizontal skin incision (Fig. 1c), the cystic eyeball was enucleated. On gross examination, anterior part of the eyeball was a cyst measuring about 15.0 mm in length filled with a yellow-colored fluid. (Fig. 1d) On pathological examination, there was a cyst with partial corneal tissue in the anterior part of the eyeball, but lens and iris were absent. To stimuli orbital development, HA (d = 21.0 mm) was implanted together with banked sclera, (Fig. 1e) after which a plastic eye shell was placed over the HA implant to maintain the conjunctival sac. Then, the incision was re-sutured. In the second step, about 1 yr later, the incision is reopened to form the upper and lower lid margins and reconstruction of the socket using full-thickness skin graft and was performed. We also employed amniotic membrane to reconstruct the fornix and a plastic eye shell was placed to maintain the conjunctival sac. The second step of surgery created a good socket to retain prosthesis (Fig. 1h). Satisfactory cosmetic results were achieved with prosthesis 1.5 years after the second step. (Fig. 1i).

## Case 2

An eleven-month-old girl was born with bilateral absent eyelid, aberrant hairline and eyebrow anomaly. (Fig. 2a) During pregnancy, his mother had no history of noticeable illness, trauma or exposure to toxic agents or radiation. The child was diagnosed as cryptophthalmos OU. Both MRI and CT scan shows an enlarged, bulging globe with anterior cyst in the right side. (Fig. 2b) To relieve consistent ocular pain in the right side, she underwent horizontal skin incision (Fig. 2c-d) and eyeball enucleation together with the cyst. On gross examination, anterior part of the eyeball was a cyst filled with a yellow-colored fluid. (Fig. 2f) The skin incision was subsequently re-sutured. At first stage, we recommended enucleation and HA implantation to stimuli orbital growth. Unfortunately, her parents preferred enucleation only and refused to receive further surgical intervention.

## Case 3

A twenty-four-year-old male patient was presented with absent eyelids and aberrant eyebrow and a horizontal skin scar can be seen in Fig. 3a. The right eye and systemic examination revealed no abnormality. During pregnancy, his mother had no history of noticeable illness, trauma or exposure to toxic agents or radiation. This patient was diagnosed to have complete cryptophthalmos OD and underwent eye enucleation at a local hospital when he was 10. On examinations of CT scan, the results revealed the left eyeball was present but the structure was disorganized (Fig. 3b). To improve cosmetic appearance, we employed banked sclera and full-thickness thigh skin graft to reconstruct the eyelid and amniotic membrane to reconstruct the fornix, which created a good socket to retain the eye shell. The prosthesis was retained well with a follow up of 2 yrs, and eyebrows grafting was performed at other hospital. (Fig. 3d).

## Discussion and conclusions

Cryptophthalmos, which means “hidden eye” in Greek, was first introduced by Zehender and he described a pediatric patient whose eyes were totally covered by skin. [4] And Manz did the autopsy on a child who died at 6 months of age, which revealed microphthalmia on one eye and ocular cyst on the other one. [4] In 1969, Francois reported a comprehensive review of the ocular and systemic condition of cryptophthalmos. He reviewed 43 cases of cryptophthalmos and divided the ophthalmic features into three categories: complete, incomplete, and abortive or congenital symblepharon. [3] Ophthalmic features of all subtypes of cryptophthalmos are summarized in Table 2.

Isolated cryptophthalmos must be differentiated from Fraser syndrome. Fraser syndrome, a rare, autosomal recessive systemic disease, was first described by Canadian geneticist Fraser in 1962. [2] And it is characterized by diverse ocular and systemic structural anomalies, the diagnosis being dependent on the presence of 2 or more major criteria in addition to 1 minor criterion, or 1 major and at least 4 minor criteria, [2, 7, 8] as outlined in Table 3. Therefore, diagnosis of Fraser syndrome must be made with caution and systemic examinations are required and all patients reported in this study didn’t meet the diagnostic standard of Fraser syndrome.

**Table 3:** Diagnostic Criteria of Fraser Syndrome^2^

Criterion	Characteristics
Major	Cryptophthalmos (complete, incomplete, or abortive form)
	Syndactyly
	Genital abnormalities (male patients: cryptorchidism, hypospadias) (female patients: uterine abnormalities, malformed fallopian tubes, clitoromegaly)
	Affected sibling
Minor	Nasal malformations
	Ear malformations
	Laryngeal malformations
	Umbilical hernia
	Renal abnormalities (dysplasia, hypoplasia, or agenesis)
	Bony abnormalities (other than syndactyly)
	Cleavage of tongue or other oral clefts
	Mental retardation

Complete cryptophthalmos is believed as an extremely rare, autosomal recessive ocular disorder. In 1986, Thomas reported a review of 124 cryptophthalmos cases which include 27 isolated cryptophthalmos and 97 syndromic cryptophthalmos. [7] They found that cryptophthalmos demonstrates equal sex distribution, occurrence in siblings, consanguinity in families with more than one affected child, and lack of vertical transmission which strongly suggesting autosomal recessive inheritance. [7] Francois and Lurie also documented several cases occurring in some families and the consanguinity between the parents in the family provides considerable evidence to suggest an autosomal recessive mode of inheritance for cryptophthalmos. [3, 9] One sibling pairs was included in our study, but their parents were not consanguineous and had no congenital abnormalities.

The exact causes of complete cryptophthalmos remain uncertain. From embryological perspective, cryptophthalmos can be explained due to failure in the movement of embryologic surface ectoderm and mesodermal nonpenetration. [2] Embryologic development of human eyelid folds occurs between 6 and 8 weeks, [10] and failure at each stage of ocular development could influences subsequent structural developments. [10, 11] Egr1, an early growth response zinc finger transcription factor with differentiation, plays a critical role in mammalian eyelid development and closure, with subsequent impact on ocular integrity. [12] Deficiency of Egr1 may result in abnormal eyelid development and closure in a genetic background-dependent manner, predisposing to a range of ocular abnormalities, including cryptophthalmos. [13] Besides, exogenous factors, such as positive history of virus infection, drug or toxic gas exposure during early pregnancy, are likely involved in the etiology of cryptophthalmos. [6]

Rehabilitation of cryptophthalmos is a challenging scenario with no agreed guidelines for its management. [1, 2, 4] Generally, the goal of surgery is to restore cosmetic and to rescue visual potential. Orbital CT scan and ocular CDI are requested to performed before surgery. If there is potential for vision, early surgery for visual rehabilitation is recommended to avoid deprivation amblyopia. However, there are no normal intraocular structure and no visual potential in most complete cryptophthalmic cases. If there the cryptophthalmic eye is painless, surgeries should be delayed until the patient is older when more tissue is available for reconstruction. [2] In case 1 and case 2, patients presented with complete cryptophthalmos and a cystic swelling arising from the orbit and suffered from persistent pain. Thus, their parents strongly request surgical intervention for help. The most crucial point of our surgical intervention is to form a conjunctiva sac. This is achieved by creating a horizontal skin incision through which a mucous membrane-covered shell conformer is placed deep to skin and subcutaneous tissue over the ocular remnant. If the eyeball is enucleated to release pain, HA implantation is suggested to promote orbital development. Then shell conformer is fitted to maintain conjunctive sac and skin incision is subsequently re-sutured. In case 1, we created a horizontal skin incision and removed the rudimentary eyeball, after which HA was implanted to stimuli orbital expansion. Besides, shell conformer is placed deep to skin and subcutaneous tissue over the HA to form conjunctiva sac.

In the second step, the incision is reopened to construct the eyelid margins 1 yr after the first step of surgery. And the lamellae of the eyelids are preferably stiffened with an ear cartilage [2] or banked sclera [6]. Mucous membrane grafting may be required to lengthen the reconstructed posterior lid lamellae. And skin may be needed to augment the anterior lamellae either as flaps or grafts. [2] Based on our experience, reconstruction and maintenance of the fornix is the most challenging task. Because the occurrence of symblepharon or shallow fornix bring about great difficulty to retain prosthesis. Various materials have been used to reconstruct the fornix, including oral mucous membrane, autologous conjunctiva, and amniotic membrane. That amniotic membrane was superior to buccal mucous membrane and hard palate in the maintenance of the fornix in cryptophthalmic repair. [14] The major advantage of amniotic is the ability to reduce inflammation and scarring while promoting epithelization. [15] Amniotic membrane has been used for conjunctival fornix reconstruction in a variety of conditions, with an overall success rate of about 50%. [14] But the disadvantage of amniotic membrane is it could be used only where there is some healthy conjunctiva. Subramanian reported adopting preputial skin graft as a great material for fornix and socket reconstruction but it could only be obtained from the male patient. [16] We employed banked sclera to reconstruct the eyelids and meanwhile autologous conjunctiva and amniotic membrane to reconstruct the fornix in case 1 and 3, which achieved desirable cosmetic and functional results. Besides, we also placed a plastic shell in the conjunctiva sac at the end of the second stage of surgery, which is helpful to maintain conjunctiva sac and prevent occurrence of symblepharon.

In the third step, retain-wearing artificial eyes were employed to improve cosmetic effect. Patients were followed up at regular intervals with the assessment of the post-operative cosmetic and functional outcomes. Meanwhile, the orbital development in pediatric patient should be highly-considered. During the follow-up, retrobulbar injection of hydrogel can be adopted to orbital expansion [17] in pediatric patients based on imagological examination. [18, 19]

In summary, oculoplastic rehabilitation in isolated complete cryptophthalmos presents specific challenges and the primary objective of surgical intervention is to improve the cosmetic appearance. The reconstruction frequently entails stepwise approaches including enucleation, reconstruction of eyelids, and further implantation of prosthesis. Additionally, orbital development should be taken into consideration in pediatric cases. To our knowledge, this is one of the largest studies to reveal several key properties of isolated complete cryptophthalmos, including clinical features, surgical interventions and long-term cosmetic outcomes.

## Data Availability

The datasets during and/or analyzed during the current study are available from the corresponding author on reasonable request.

## Abbreviations

CDI: color doppler imaging
CT: computed tomography
EGR: early growth response
HA: hydroxyapatite
MRI: magnetic resonance imaging
